# Nanoplastic production procedure for scientific purposes: PP, PVC, PE-LD, PE-HD, and PS

**DOI:** 10.1016/j.heliyon.2023.e18387

**Published:** 2023-07-17

**Authors:** Patricia Merdy, Floriane Delpy, Adrien Bonneau, Sylvie Villain, Lucian Iordachescu, Jes Vollertsen, Yves Lucas

**Affiliations:** a Université de Toulon, Aix Marseille University, CNRS, IM2NP, 83041, Toulon, France; b Aalborg University, Department of the Built Environment, Thomas Manns Vej 23, 9220, Aalborg, Denmark

**Keywords:** Nanoplastics, Dissolution-precipitation, Nanoparticles production

## Abstract

Studies on the environmental impact of nanoplastics face challenges in plastic analysis and a scarcity of nanoplastic materials necessary for the development of analytical techniques and experiments on biota impact. Here we provide detailed procedures for obtaining nanoparticles suspended in water for the most commonly used polymers: Polypropylene (PP), Polyvinylchloride (PVC), Low- and High-Density Polyethylene (PE-LD, PE-HD), and Polystyrene (PS). We dissolved larger size material to reprecipitate nanoparticles. For all plastic types, we obtained nanoparticles with a size between 50 and 300 nm, and a mainly spherical morphology. We verified that no irreversible agglomeration or coalescence of the particles occurred after 5 days of storage. The concentrations obtained in the final carrier solution were of the order of 10^9^ particles mL^−1^. To prevent the persistence of reagents in the final carrier solution, a filtration step was implemented at the end of the process. The method proved unsuitable for Polyethylene Terephthalate (PET).

## 1 Introduction

The great persistence of plastic polymers in the environment makes them ubiquitous pollutants throughout the world. They represent between 60% and 80% of total marine debris, and soil contamination is estimated to pose a similar problem [1] The main size fraction of these particles is smaller than 10 mm [2] and the micro- and nanoplastics released are of an appropriate size to be ingested by the biota [3] leading to bioconcentration, bioaccumulation and disturbance of organisms [4].

According to the European Chemicals Agency which proposed in 2019 a regulatory definition for microplastics under the REACH legislation, microplastics refer to particles of solid polymer that range in size from 1 nm to 5 mm, as well as fibres with a length-to-diameter ratio greater than 3 and a length ranging from 3 nm to 15 mm. According to International Organization for Standardization (ISO), the nanoscale corresponds typically to one or more dimensions below 100 nm [5]. Nevertheless, in the scientific community, different methods are used to define size ranges. Here we followed the recommendations of Hartmann et al. [6] who proposed, after an exhaustive critical review, to categorize everything that is less than 1000 nm as nanoplastic.

In the marine ecosystem, it has been shown that the impact of the micro-particulate fraction on the various trophic levels causes significant disturbances (inflammation, poisoning, endocrine disruption, etc) [7]. However, studies concerning the <5 μm fraction remain rare, and therefore the impact of nanoplastics on biota is still very poorly assessed. Studies are rare because of two major issues, (i) the lack of reference plastic material and (ii) detection problems at this size scale. However, the smaller size of these particles enables their easier penetration into cells: they have the ability to cross epithelial barriers to pass into the general bloodstream, reach internal organs [8] and end up in the nuclei of cells where they are likely to be genotoxic [9]. PS (polystyrene) nanoparticles are commonly employed in ecotoxicological studies due to their widespread commercial availability with a specific size distribution. The other plastic types are more difficult to produce at a nanoscale level.

In addition to assessing toxicity, there are other requirements for nanoscale plastic materials, specifically related to the development of analytical techniques used for the identification and quantification of nanoplastics. Among the most recent and challenging developments, we can mention fluorescent labelling [10] or enhanced Raman spectroscopy [11]. These recent developments are applied to various types of sample: marine or fresh water, soils, atmospheric compartments, wastewater from wastewater treatment plants before and after treatment to assess the purification process.

In this context, it is imperative to be able to produce reference material with a well-defined size and morphology. Despite a comprehensive description of the processes and factors used in the different methods of preparing micro- and nanoplastics [12], there is still a scarcity of studies that provide complete information on the operational methods used for their elaboration [[13], [14], [15], [16]]. The produced plastic nanoparticles must meet several criteria: purity, a size distribution adapted to the intended use, a controlled morphology, and good reproducibility of their synthesis. Literature searches indicate different ways of approaching the problem. Either the choice is a top-bottom method which consists of transforming a coarse material into homogeneous nanoparticles, or the choice falls on a bottom-up method in order to polymerize plastic monomers or to precipitate polymer chains into particles of controlled sizes, or the coarse plastic is submitted to physical mechanical and thermal stresses in order to reach nanometric sizes.

In several studies, mechanical grinding has been explored as a top-bottom procedure to assess the changes in polymer properties during dry fine grinding under cryogenic conditions. PVA (polyvinylacetate), PE (polyethylene) and PS were studied in Molina-Boisseau et al. [15,17] who showed that the morphology of the fragments obtained varied according to the nature of the initial material and the specific operating parameters. They did not obtain nanoparticles but microparticles of plastics ranging from 10 to 200 μm. Microplastics assumed to be similar to those found in the environment were produced and studied by two different methods in Kefer et al. [18]: on the one hand, a top-down grinding method, on the other hand, a bottom-up method by precipitation of dissolved polymers. The particles produced by grinding exhibited wide size distribution, cracks and irregular shape, while the particles produced by dissolution-precipitation were highly porous with 15–60 μm size. Still, nanosize particles were not obtained. In their study, Schmidt et al. [19] successfully obtained particles of PS and polyetheretherketone (PEEK) with sizes smaller than 5 μm through a wet grinding procedure. This process involved the use of organic solvents (ethanol, hexane) at a low temperature of −80 °C, while avoiding the need for liquid nitrogen. However, despite this notable advancement in particle size reduction, the resulting sizes still fall within the micrometric range.

Recently, some studies managed to reach nanosized plastic production. In their work, Balakrishnan et al. [14] devised a method that involves nanoprecipitation of a PE solution in toluene, using chemical or bio-sourced surfactants. Similarly, Rodriguez-Hernandez et al. [20] utilized nanoprecipitation to generate polyethylene terephthalate (PET) nanoparticles from PET pellets. Pessoni et al. [21] synthesized nanoplastics with various surface functionalities through soap free emulsion polymerization. They obtained monodisperse polymers. Following a top-down approach, Magri et al. [22] used laser ablation to obtain PET nanoplastics of 100 nm average size. Astner et al. [23] employed mechanical degradation techniques to generate micro- and nanoplastics from a mulch film composed of polybutyrate adipate-*co*-terephthalate and low-density PE (PE-LD) polymers. Similarly, El Hadri et al. [24] utilized mechanical degradation as a means to simulate environmental processes.

This present study proposes an easy route for producing six different plastic nanoparticles in suspension, starting from commercially available larger sized beads, by modifying and extending to PE-LD, PE-HD, PVC, PS and PP the PE procedure proposed by Balakrishnan et al. for PE [14].

## 2 Materials and methods

### 2.1 Chemicals

**Initial crude material.** The plastic materials were supplied by GoodFellow (Lille, France). The initial bead sizes were different according to the plastic type: PE-LD, 300 μm (ref. ET316031); PE-HD, from 2 to 4 mm (ref. ET326310); PVC, 250 μm (ref. CV316010); PS, 900 μm (ref. ST316003); PP, 5 mm (ref. PP306312); PET, 300 μm (ref. ES306030). The toluene, NaOH and Tween60 or Tween80 surfactants were purchased from Sigma-Aldrich, Merk KGaA, Darmstadt, Germany. Tween60 and Tween80 are the brand names for polysorbate 60 or polysorbate 80, both known as non-ionic surfactants.

**Preparation of the initial solutions**. The Tween80 surfactant (oil-in-water emulsifier) initial solution of 1% (m/m) concentration was prepared by diluting 0.2 g of Tween80 in 20 g of toluene at 20 °C. A 100 mL volume of sodium chloride solution at 35 g L^−1^ was prepared in parallel. Both solutions were kept at 3 °C and stored in the dark.

### 2.2 Experimental procedure

#### 2.2.1 PE-LD, PE-HD, PVC and PP

**Fig. 1:**
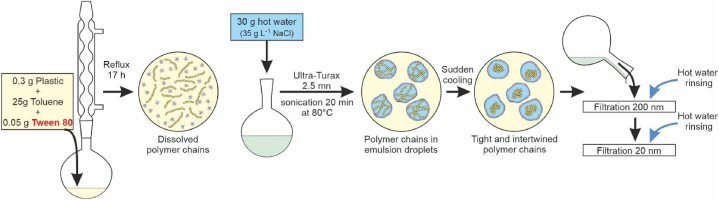
Sketch of the experimental procedure for PE-LD, PE-HD, PVC and PP.

The experimental procedure (Fig. 1) involved the preparation of nanoplastic emulsions from coarse beads using a surfactant (Tween80, an oil-in-water emulsifier), saltwater, and a solvent. Among the various solvents considered (benzene, chloroform, dimethylformamide, tetrachloromethane, trichloroethylene, toluene, xylene, etc.), toluene emerged as the most suitable solvent for each of the polymers considered. This choice was based on its solvent properties as well as safety, health, and environmental factors. The reflux system was set up at 80 °C for 17 h, followed by strong mechanical stirring for 180 s using an Ultra-Turrax homogenizer (IKA T18 Digital equipped with a S18N-10G rotor-stator dispersion unit) to create a toluene-in-water microemulsion. Nanoplastic precipitation within the emulsion microdroplets occurred upon cooling after sonication. Filtration using two successive alumina filters (Whatman Anodisc 47) with cut-off thresholds of 0.2 μm and 0.02 μm was performed to control the size of the obtained material. Excess reagents were removed by rinsing with 200 mL of hot distilled water (60 °C). Lastly, the nanoplastic material was collected in suspension by sonicating the filter in a solvent, such as water.

As depicted in Fig. 1 and based on the interpretations given in Balakrishnan et al. [14] this procedure is based orelies on several steps: (1) the dissolution of polymer chains in a solvent that does not degrade or break them, (2) the isolation of a limited number of chains within the solvent droplets of the emulsion, (3) the precipitation of the chains within each droplet through entanglement and intertwining upon cooling. The size of the nanoparticle is therefore determined by the number of chains contained within a droplet.

The modifications we made to the procedure proposed by Balakrishnan et al. [14] gave satisfactory results on the four tested plastics. We found it preferable to use Tween80 rather than Tween60, as the latter tending to crystallize on the glassware during the experimental procedure. The use of salt water improved emulsion stability, likely by reducing droplets coalescence [25]. Compared to lyophilization, the filtration steps at the end of the experimental protocol facilitated the removal of remaining reagents and constrained the nanometric size.

#### 2.2.2 PS

Since polystyrene is not soluble in toluene, we used acetone instead toluene as the solvent, with the same protocol than for PE-LD, PE-HD, PVC and PP. Acetone is miscible in water, but PS chains are not, resulting in an anti-solvent precipitation [12,26], the surfactant being able to change the local chain density [27].

#### 2.2.3 PET

PET is not soluble in most common organic solvents and the most commonly used solvent for its solubilization are toxic and expensive. Although a simple procedure for producing PET nanoparticles using trifluoroacetic acid, which is relatively non-toxic, has been proposed elsewhere [25] we tested a procedure based on the alkaline hydrolysis of PET using NaOH. NaOH dissolves PET by cleaving the ester bond but, without external catalyst, the PET chains attack is complete only at high pressure and temperature [28]. It is likely, however, that a hydraulitic degradation of the PET chains occurs, resulting in a change in the molecular weight distribution of the chains. Here we used a hot (80 °C) NaOH solution at atmospheric pressure to chemically dissolve the PET. The addition of Tween80 was maintained to promote the coalescence of the polymer chains by salting-out effect [9] during the neutralization of the base and the cooling.

### 2.3 Characterization

#### 2.3.1 Size analysis

Nanoparticle tracking analysis (NTA) was performed on the obtained suspensions with a Nanosight NS-500 instrument (Malvern Panalytical, Malvern, UK). The laser wavelength was 405 nm. This technique involves the laser light scattering with an optical microscope, where the camera records the scattered light emitted by individual particles that are undergoing Brownian motion. The software NTA 2.3 was used to process the video motion of particles and to calculate their sphere-equivalent hydrodynamic diameter using the Stokes-Einstein equation. In the best of cases (gold nanoparticles for example) this instrument can determine the size of particles ranging from 10 to 2000 nm. With plastic particles, peaks below 40 nm are unreliable and may be artifacts. The instrument measurement conditions were optimised with commercial, calibrated, 50 and 200 nm size polystyrene suspensions (Malvern Panalytical, Malvern, UK). Five video captures of 60-s each were taken of each nanoplastic sample produced, between 1000 and 2000 valid tracks were obtained for each analysis.

#### 2.3.2 Nanoplastic chemical fingerprint

For IR analysis, a Cary 620 FTIR microscope (Agilent Technologies, Santa Clara, CA, USA) coupled with a Cary 670 IR spectroscope was utilized to verify the composition of the nanoplastic suspensions. The setup included a 15× Cassegrain objective and a 128 × 128 Mercury Cadmium Telluride (MCT) FPA detector, providing a pixel resolution of 5.5 μm. A 100 μl aliquot was deposited onto a Ø 13 mm zinc selenide window and allowed to dry at 50 °C. This process was repeated until suitable particle agglomeration was achieved. IR scans were conducted from 3750 to 950 cm^−1^ with 8 cm^−1^ steps. In transmission mode, 30 co-added scans were performed, while 120 co-scans were used for background measurement. Multiple areas measuring 1408 μm × 1408 μm were scanned until a spectrum for each polymer was obtained.

#### 2.3.3 Particle morphology

Samples were visualized by Scanning Electron Microscopy (SEM) on a Zeiss supra 40 V P SEM (Carl Zeiss Microscopy GmbH, Iena, Germany), fitted with a GEMINI column, which made it possible to work in Inlens mode. It was equipped with an energy dispersive spectroscopy (EDS) microanalysis system. The nanoparticle solution samples were deposited on copper, silicon and/or platinum substrates (one drop deposit). They were not metallized before observation. The areas of interest were first located in backscattered electron mode.

## 3 Results and discussion

### 3.1 Size distribution

**Fig. 2:**
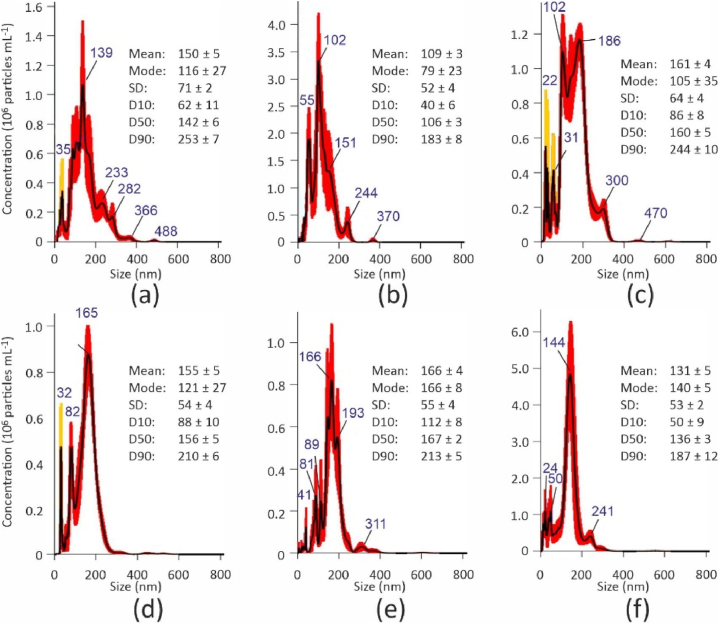
NTA particle size distributions of nanoplastic suspensions. (a) PE-HD; (b) PP; (c) PS; (d) PVC; (e) PE-LD; (f) PET. Statistical values in nm. SD: standard deviation; D10, D50 and D90 represent the size below which 10%, 50% or 90% of all particles are found. The red marks indicate standard deviation obtained from five runs. Blue value indicates the particle size at the peak. Orange peaks corresponds to size <40 nm. (For interpretation of the references to color in this figure legend, the reader is referred to the Web version of this article.)

**Fig. 3:**
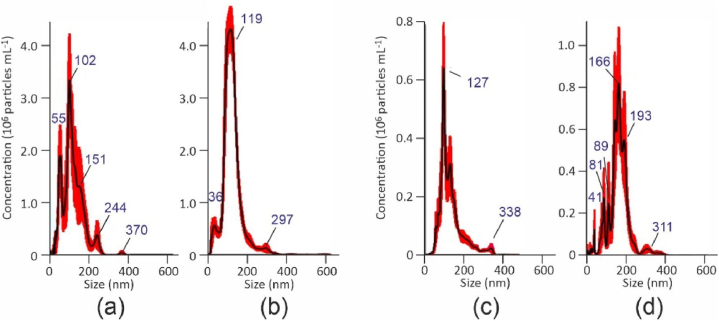
Example of difference between NTA particle size distribution for two different synthesis sessions. (a) PP, session 1; (b) PP, session 2; (c) PE-LD, session 1; (d) PE-LD, session 2.

The yields obtained for all polymers were approximately 5% of the initial raw plastic material. The concentrations achieved after the experiments were approximately 2 × 10^9^ particles mL^−1^. Examples of the size distributions can be observed in Fig. 2. While most nanoparticles were found between 50 and 300 nm for all types of plastic, their distributions varied among these types (Fig. 2) and across different synthesis sessions (Fig. 3). PE-LD and PET exhibited distributions that closely resembled a unimodal distribution, whereas the other plastics showed additional peaks for sizes below 100 nm. However, it is worth noting that peaks corresponding to sizes smaller than 40 nm (indicated by the orange color in Fig. 2) might be artifacts resulting from the NTA apparatus. These discrete distributions potentially suggest that the different peaks correspond to small aggregates of smaller particles. Insufficient control over emulsification, cooling kinetics and the duration before the filtration step can account for variations in the results obtained from various synthetic sessions.

### 3.2 Stability of the suspension over time

**Fig. 4:**
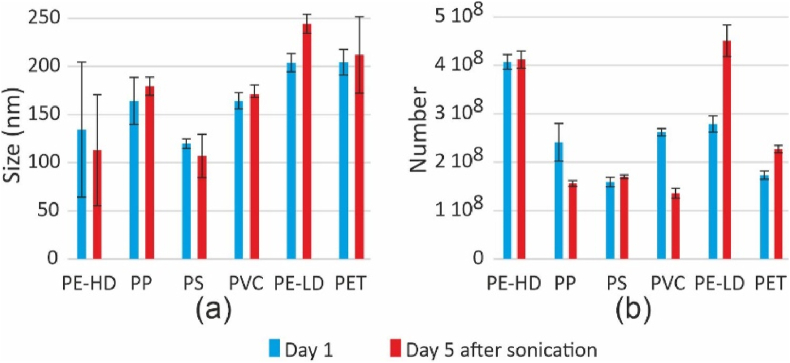
NTA measurements immediately after production (blue) and after 5-days storage (red). (a) Size distribution (mode) and (b) particle concentration. Error bars indicate ± SD. (For interpretation of the references to color in this figure legend, the reader is referred to the Web version of this article)

A relatively small quantity of particles had a size greater than 200 nm, surpassing the membrane cut-off size. This suggests that some particles tended to agglomerate over time. Therefore, we investigated the evolution of the suspensions over the course of a week. NTA analysis was conducted immediately after particle production, followed by analysis after 5 days of storage of the suspension at 4 °C with subsequent sonication for a few minutes. In this series of samples, the concentration of nanoparticles was approximately 10^8^ particles mL^−1^. The results are given in Fig. 4. Most plastic types do not exhibit significant differences in particle size following storage. Only PE-LD demonstrated a slight increase in size, from 204 to 244 nm. The particle count exhibited certain fluctuations, highlighting the significance of recognizing the sensitivity of particle aggregation and fragmentation. The presence of small particles merging into larger ones or, conversely, larger particles breaking down into smaller ones can greatly impact the overall particle count. Even a minor alteration in the quantity of large particles can lead to a substantial fluctuation in the count of small particles.

**Fig. 5:**
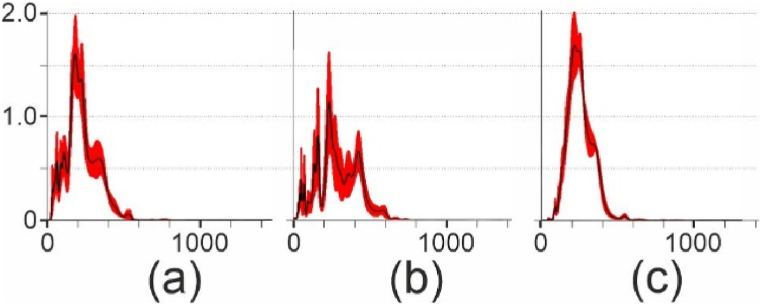
NTA size distribution of PE-LD nanoparticles suspension: (a) immediately after production; (b) after 5 days before sonication; (c) after 5 days after sonication.

Fig. 5 provides an illustrative example of the particle size distribution immediately after synthesis (5 A), as well as after a five-day period both before (5 B) and after (5C) sonication. The aging process over the five-day period resulted in aggregation of the particles, leading to larger particle sizes. However, sonication effectively dispersed these aggregate, resulting in a distribution similar to the pre-aging distribution. Notably, a surprising observation was the absence of the finest particles after sonication. Further investigations are needed to determine the cause of these variations. Nonetheless, it can be concluded that nanoparticle suspension can be stored successfully. As an optional long-term storage measure, the addition of NaN_3_ to a final concentration of 10^−2.5^ mol L^−1^ may be considered to prevent potential biological contamination, provided that the corresponding ionic strength (10^−2.5^ mol L^−1^ in the absence of other ionic solute) does not lead to alterations in the nanoplastics.

### 3.3 Particle morphology

**Fig. 6:**
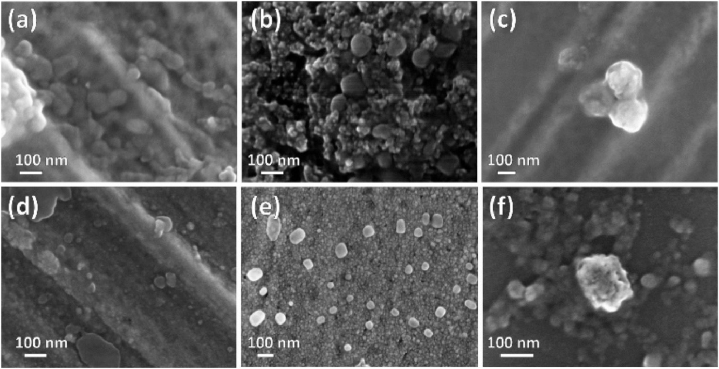
SEM views of the nanoplastic particles. (a) *P*-HD; (b) PP; (c) PS; (d) PVC; (e) PE-LD; (f) PET.

SEM images of the produced particles are presented in Fig. 6. In all samples, the grains exhibited a relatively spherical shape, with the presence of agglomerates of varying size. For example, Fig. 6 shows a 3-particle agglomerate visible in the PS view. Particle sizes of individual particles, discarding agglomerates, obtained from statistical measurements through image analysis were 40 nm (PE-LD and PET), 50 nm (PVC), 70 nm (PE-HD and PP) and 200 nm (PS). Except for PP and PS, these values are 2–3 times smaller than those found with the NTA analysis, consistent with the existence of agglomerates. Particle sizes are determined based on the Brownian motion exhibited by particles. By performing calculations, it is possible to estimate the number of small individual particles that would agglomerate to form a larger particle with an apparent size. The calculation, based on the observed size of small individual particles in the SEM images, revealed that the modes indicated in Fig. 2 would correspond to agglomerates consisting of 5, 14, 71 and 43 small particles for PE-HD, PVC, PE-LD and PET, respectively. These agglomerates appear to be more stable than the aggregates formed during aging and do not separate upon sonication.

### 3.4 Particle IR analysis

In order to check that the experimental procedure led to the expected plastic without deterioration, we used the focal plane array-based μFT-IR imaging technique. The aggregation of the nanoplastics during drying made it possible to acquire good quality spectra with the μFTIR instrument. The obtained IR maps were processed using the siMPle software [29], where each spectrum was correlated to a reference library composed of more than 100 spectra of synthetic and natural materials.

The spectra with the best quality match underwent a second verification using OMINC 8.3 software (Thermo Fisher Scientific Inc, MA, USA) which also used a vast library of spectra [30]. PP, PVC, PE-HD, PE-LD and PS were successfully identified by both softwares (Fig. 6). It was not possible to acquire an IR spectrum of the sample containing PET, which may be related to hydrolytic degradation of the polymer chains, as noted above (section 2.2.3).

**Fig. 7:**
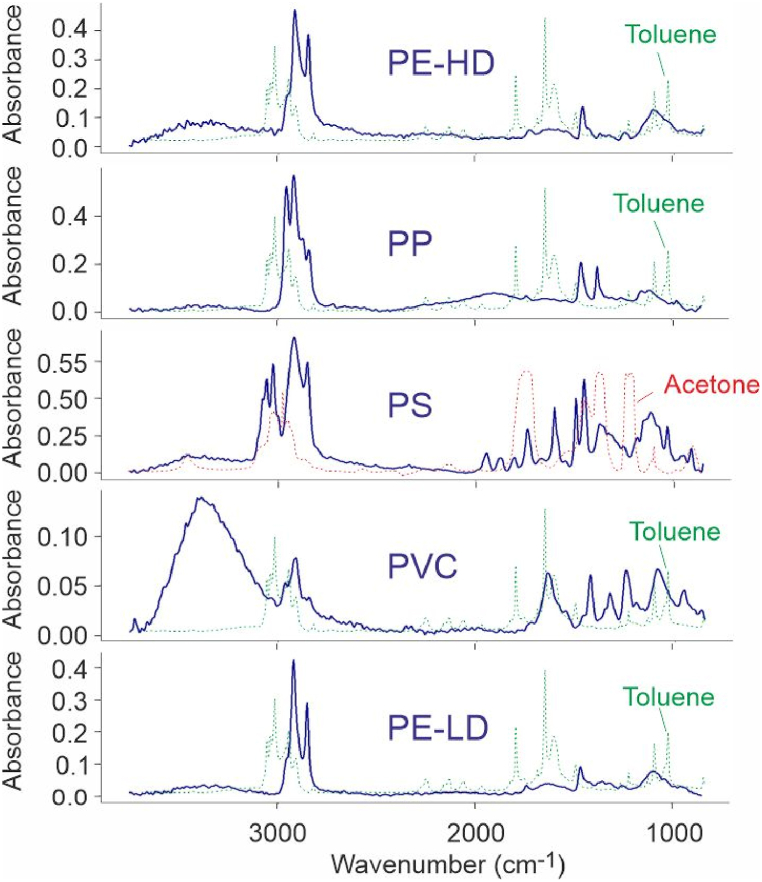
FTIR analysis of the 5 nanoplastic suspensions: PP, PVC, PE-HD, PE-LD, PS. Superimposed solvent FTIR spectra showed their absence or low residual content after the last production step.

We rinsed the solvent and the surfactant with hot water under vacuum filtration. The solvent peaks did not appear on the FTIR spectra (Fig. 7). The sensitivity limit of the technique does not ensure the absence of residual amounts of solvent or surfactant of less than 5%. However, considering that the solubility of Tween80 and acetone in water is very high, and that of toluene in water at 60 °C is 1 g L^−1^ [31], it is very probable that the surfactant and the solvent remain present only in traces at most.

## 4 Conclusions

For the five most commonly used types of plastic (PE-HD, PP, PS, PVC, and PE-LD), the experimental protocols described above enable the production of nanoparticles between 50 and 300 nm. These protocols effectively prevent the persistence of problematic reagents such as salt or surfactants in the carrier solution. The concentrations obtained are in the order of 10^9^ particles mL^−1^ and particles exhibit a predominantly spherical morphology. This allows a self-production of nanoparticles that serves as a fundamental step in material production for studies focused on nanoplastic analysis techniques or to the impact of nanoplastics on biological targets. However, for PET, the application of the method did not yield satisfactory results.

Commercial PS was analyzed by NTA and showed narrower size distribution, for both the 50 nm standard and the 200 nm standard. Nonetheless, our procedure provides an important initial step in obtaining a wide range of nano-size plastic materials, offering a valuable opportunity for researchers who were previously limited by the lack of commercially available materials.

The next steps could involve precise control over particle size by adjusting the relative quantity of the reagents, the reaction time, and cooling parameters Additionally, the development of a continuous flow production technique could be explored to obtain large quantities of nanoparticles. We propose that the implementation of nano tangential flow filtration technique could further narrow the size distribution after the final production step proposed in this work.

## Author contribution statement

Patricia Merdy: Conceived and designed the experiments; Performed the experiments; Analyzed and interpreted the data; Contributed reagents, materials, analysis tools or data; Wrote the paper.

Adrien Bonneau: Performed the experiments.

Floriane Delpy, Sylvie Villain & Lucian Ioradeschu: Performed the experiments; Analyzed and interpreted the data.

Jes Vollertsen: Contributed reagents, materials, analysis tools or data; Analyzed and interpreted the data.

Yves Lucas: Analyzed and interpreted the data; Wrote the paper.

## Data availability statement

Data will be made available on request.

## Declaration of generative AI and AI-assisted technologies in the writing process

During the preparation of this work the authors used CHAT-GPT in order to check that the English text was grammatically correct. After using this tool, the authors reviewed and edited the content as needed and take full responsibility for the content of the publication.

## Declaration of competing interest

The authors declare that they have no known competing financial interests or personal relationships that could have appeared to influence the work reported in this paper.
